# Promotion of vascular integrity in sepsis through modulation of bioactive adrenomedullin and dipeptidyl peptidase 3

**DOI:** 10.1111/joim.13220

**Published:** 2020-12-30

**Authors:** D. van Lier, M. Kox, P. Pickkers

**Affiliations:** ^1^ From the Department of Intensive Care Medicine and Radboud Center for Infectious Diseases (RCI) Radboud University Medical Center Nijmegen The Netherlands

**Keywords:** cardiovascular regulation, endothelial function, sepsis, vascular disease

## Abstract

Sepsis represents one of the major medical challenges of the 21st century. Despite substantial improvements in the knowledge on pathophysiological mechanisms, this has so far not translated into novel adjuvant treatment strategies for sepsis. In sepsis, both vascular tone and vascular integrity are compromised, and contribute to the development of shock, which is strongly related to the development of organ dysfunction and mortality. In this review, we focus on dipeptidyl peptidase 3 (DPP3) and adrenomedullin (ADM), two molecules that act on the vasculature and are involved in the pathophysiology of sepsis and septic shock. DPP3 is an ubiquitous cytosolic enzyme involved in the degradation of several important signalling molecules essential for regulation of vascular tone, including angiotensin II. ADM is a key hormone involved in the regulation of vascular tone and endothelial barrier function. Previous studies have shown that circulating concentrations of both DPP3 and ADM are independently associated with the development of organ failure and adverse outcome in sepsis. We now discuss new evidence illustrating that these molecules indeed represent two distinct pathways involved in the development of septic shock. Recently, both ADM‐enhancing therapies aimed at improving endothelial barrier function and vascular tone and DPP3‐blocking therapies aimed at restoring systemic angiotensin responses have been shown to improve outcome in various preclinical sepsis models. Given the current lack of effective adjuvant therapies in sepsis, additional research on the therapeutic application of these peptides in humans is highly warranted.

## Graphical abstract

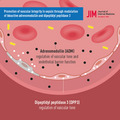

## Introduction

Despite advances in medical care, sepsis remains a major health problem of the 21st century, with a high mortality and an ever‐increasing incidence [1]. Sepsis is now viewed as an inflammatory disorder, in which a dysregulated host response to infection results in life‐threatening organ dysfunction [2]. In septic shock, the most severe form of sepsis, profound underlying circulatory, cellular and metabolic abnormalities, is associated with an even greater risk of mortality [2, 3]. Septic shock is characterized by increased lactate levels, as well as a necessity for vasopressor therapy to maintain adequate blood pressure and organ perfusion, despite adequate fluid resuscitation [3, 4].

Sepsis consists of a complex, multifaceted pathogenesis, in which the sum of many harmful and protective pathways results in the observed clinical condition [5]. During sepsis, a host response is mounted after pathogen‐associated molecular patterns (PAMPs) are recognized by highly conserved pattern recognition receptors (PRRs) present on immune cells [4, 6]. Activation of these receptors leads to the activation of multiple inflammatory pathways including leucocyte and complement activation, the release of pro‐inflammatory cytokines, reactive oxygen species and damage‐associated molecular patterns (DAMPs) [6, 7]. All these factors ultimately contribute to the development of organ failure, which is the key determinant of sepsis mortality [4].

During sepsis, vascular tone and integrity are compromised, with both factors contributing to the development of shock. Endothelial dysfunction is one of the major hallmarks of sepsis [8]. A profound inflammatory response causes disturbed endothelial cell signalling and endothelial cell death [9, 10]. Subsequent loss of endothelial barrier integrity results in extravasation of fluids and molecules, causing oedema and the loss of intravascular volume. Ultimately, this leads to a decrease in blood pressure, which further contributes to organ failure [4, 8, 9].

Although knowledge on the molecular mechanisms causing sepsis has substantially improved, treatment strategies have remained virtually unchanged for decades, implying that the gap between fundamental knowledge and clinical application has only widened. Treatment consists of adequate and timely antimicrobial therapy, source control, supportive therapies including fluid resuscitation and vasopressor therapy to maintain vascular tone and organ support interventions such as mechanical ventilation and renal replacement therapy [9]. The majority of conducted sepsis trials investigating possible adjuvant treatments focused on attenuating pro‐inflammatory responses [11]. Unfortunately, none of these therapies improved clinical outcome, with some even resulting in increased mortality [12, 13, 14]. This lack of therapeutic benefit observed in clinical sepsis trials can be partially explained by considerable patient heterogeneity, resulting from inter‐individual differences in comorbidity, comedication, source of infection, causative pathogens and timing of onset of the inflammatory response [4, 15]. This heterogeneity impedes evaluation of pathophysiological mechanisms and hampers accurate assessment of pharmacological interventions [16].

Taken together, it is clear that there is still an unmet need for novel treatment strategies for sepsis. Targeted interventions aimed at improving endothelial barrier function and vascular tone may prove highly relevant in this regard [8]. In this review, we focus on dipeptidyl peptidase 3 (DPP3), an ubiquitous cytosolic enzyme involved in the degradation of several important signalling molecules essential for regulation of vascular tone, including angiotensin II [17, 18], and adrenomedullin (ADM), a key hormone involved in the regulation of vascular tone and endothelial barrier function [19]. We describe the general vascular properties of DPP3 and ADM and provide an overview of the current understanding of the different roles of these molecules in sepsis and septic shock. Furthermore, we discuss the potential of DPP3‐ and ADM‐targeted treatments for sepsis patients, as well as the implications of a completed biomarker‐guided trial incorporating ADM measurements on future sepsis trial designs.

## Dipeptidyl peptidase 3

DPP3 was the third enzyme in the dipeptidyl peptidase group to be identified when it was first isolated from bovine pituitary tissue more than half a century ago [20]. DPP3 is a zinc‐dependent metallopeptidase capable of hydrolysing a broad spectrum of oligopeptides between three and ten amino acids in length [18]. DPP3 has been implicated in blood pressure regulation [21], inflammation [22] and pain regulation [23, 24] through its capability to hydrolyse and thus inactivate bioactive peptides such as angiotensins, enkephalins and endorphins [18].

DPP3 is ubiquitously expressed in a range of tissues including erythrocytes, leucocytes, lung, heart, kidney, intestines, skeletal muscle, skin, brain, liver and spleen [22, 25, 26, 27, 28, 29, 30, 31, 32]. Whilst DPP3 is classified as a primary cytosolic enzyme [26, 33], membrane‐bound forms of DPP3 have been described in neutrophils, brain tissue and different visceral organs [22, 34]. More recently, specific immunoassays for the detection of DPP3 concentration and enzyme activity in plasma have been developed, which demonstrated the constitutive presence of DPP3 in the circulation (coined cDPP3 for circulating DPP3) [35].

DPP3 exercises its enzymatic function by cleaving a dipeptide fragment from the N‐terminus of its substrates [36]. Its catalytic zinc‐binding domain closely resembles that of other notable but structurally unrelated metallopeptidases such as neprilysin and thermolysin [36]. The catalytic domain of DPP3 is highly preserved between species, underlining its function as an enzyme of biological significance [18]. Of its known substrates, tripeptides are only poorly hydrolysed [37], whilst peptides containing more than ten amino acids cannot be cleaved by DPP3 [20, 34].

cDPP3 has a half‐life of approximately 70 min [35]. The mechanisms through which DPP3 is cleared from the circulation are unknown. Nevertheless, studies on the clearance kinetics of other enzymes suggest that primary endocytosis in the liver followed by further processing in lysosomes [38] could be responsible. Similarly, it is currently unclear to what extent kidney and/or liver dysfunction influence cDPP3 clearance kinetics.

## DPP3 as a depressant of the cardiovascular system

The renin–angiotensin–aldosterone system (RAAS) plays a vital role in the regulation of cardiovascular system homeostasis [39]. The primary effector molecule of this system, angiotensin II, affects the function of virtually all organs, and both beneficial and pathological effects have been reported [39, 40, 41]. Acute changes in angiotensin II mainly serve to raise blood pressure through increases in sympathetic tone, endogenous catecholamine and vasopressin release, as well as direct stimulation of vascular smooth muscle cells (VSMCs) [39, 41]. Angiotensin II is also essential to maintain glomerular filtration, especially during periods of reduced renal perfusion [42]. Following chronic stimulation with angiotensin II, the opposite is observed, as this is associated with adverse vascular and cardiac remodelling through induction of hypertrophy as well as fibrosis of VSMCs and cardiomyocytes [39, 40, 41].

Until recently, the interplay between DPP3 and RAAS was only scarcely studied [18, 43]. Multiple studies had already pointed out the putative rapid angiotensin‐scavenging properties of DPP3 based on *in vitro* experiments [18]. Angiotensin II, angiotensin III, angiotensin IV, angiotensin 1‐5 and angiotensin 1‐7 were all found to be effectively hydrolysed by DPP3 [17, 18, 44], with angiotensin IV (six amino acids in length) being hydrolysed ten times faster than angiotensin II (eight amino acids in length) [21]. However, as reliable assays to measure cDPP3 were not available, these findings could not be confirmed *in vivo*. Following the recent development of cDPP3 luminometric immunoassays, which also demonstrated the constitutive cDPP3 presence in healthy humans, interest in the field was revitalized [35].

Whereas cDPP3 levels are low in healthy volunteers [35], high cDPP3 concentrations are found in sepsis, septic shock, cardiogenic shock and burn victims exhibiting vasodilatory shock syndrome [35, 45, 46, 47]. In these patient cohorts, admission cDPP3 levels were associated with higher organ dysfunction scores, the development of myocardial dysfunction, refractory shock, acute kidney injury and increased short‐term mortality [35, 45, 46, 47]. Interestingly, a decrease in cDPP3 following treatment was associated with less subsequent organ support requirements and lower mortality in all of these conditions [45].

**Fig. 1:**
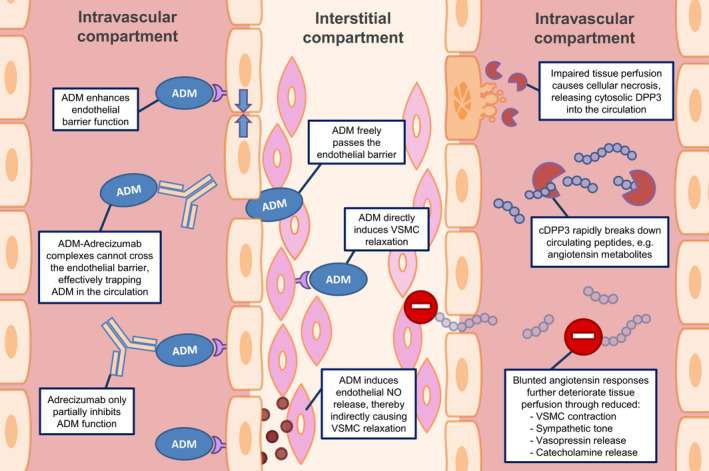
Overview on the effects of adrenomedullin (left part) and circulating dipeptidyl peptidase 3 (right part) on vascular function and the mode of action of the non‐neutralizing adrenomedullin antibody Adrecizumab. ADM = adrenomedullin, VSMC = vascular smooth muscle cell, NO = nitric oxide, cDPP3 = circulating dipeptidyl peptidase 3.

Based on these clinical associations combined with the known short half‐life and primary cytosolic localization of DPP3 [26, 35], it was hypothesized that high levels of cDPP3, despite adequate supportive treatment, represent a state of ongoing cell death (necrosis) [45] and release of cytosolic DPP3 into the circulation. During shock, upregulation of angiotensin II is a physiologic and potentially life‐saving response aimed at maintaining adequate tissue perfusion [41, 48]. Since the uncontrolled release of DPP3 into the circulation is able to effectively cleave angiotensin II, DPP3 might represent a novel factor contributing to the deterioration of vascular tone in different shock conditions [18]. An overview of the effects of cDPP3 is presented in Fig. 1 and Table 1.

**Table 1:** Overview on the biological, pathophysiological, prognostic and therapeutic properties of ADM and DPP3 in sepsis

	ADM	DPP3
General characteristics	‐ Ubiquitous signalling peptide [25] ‐ Exercise effects through stimulation of ADM1 and ADM2 receptors [58] ‐ Low circulating levels in healthy subjects [59]	‐ Ubiquitous (primarily) cytosolic enzyme [25, 33] ‐ Degrades bioactive peptides, most notably angiotensins, enkephalins and endorphins [18] ‐ Low circulating levels in healthy subjects [35]
Biological effects	‐ ADM present in the interstitial space reduces vascular tone through VSMC relaxation [80, 89] ‐ ADM present in the circulation stabilizes the endothelial barrier [108, 109]	‐ Reduces vascular tone by degrading circulating angiotensin II [21] ‐ Cytosolic terminal protein turnover [18]
Metabolism	‐ Membrane‐bound proteases [73] ‐ Degradation of ADM‐ADM receptor complexes [76, 77, 78]	‐ Unknown; based on studies of similar enzymes, endocytosis in the liver followed by processing in lysosomes is likely [38]
Prognostic properties in sepsis	*High circulating levels are associated with:* ‐ Disease severity [110, 111, 112, 116] ‐ Organ dysfunction (vasopressor therapy, acute kidney injury) [110, 116] ‐ Short‐term mortality [110, 111, 112, 115, 116]	*High circulating levels are associated with:* ‐ Disease severity [35, 45] ‐ Organ dysfunction (vasopressor therapy, acute kidney injury, myocardial depression) [45, 46, 47] ‐ Short‐term mortality [35, 45, 46, 47]
Therapeutic properties in animal models	ADM administration: ‐ Improves survival, reduces endothelial hyperpermeability and attenuates end‐organ injury in animal sepsis models [119, 120, 122, 123, 124] ADM non‐neutralizing antibodies: ‐ Improve survival, reduce vasopressor requirements and attenuate end‐organ injury in animal sepsis models [131, 132, 133] ADM‐neutralizing antibodies: ‐ Do not improve outcome in an animal sepsis model [131]	DPP3 administration: ‐ Rapidly provokes left ventricular dysfunction in healthy mice [46] ‐ Reduces blood pressure and attenuates adverse cardiac remodelling in a murine hypertension model [21] DPP3‐neutralizing antibodies: ‐ Improve survival, and reduce myocardial dysfunction and cardiac oxidative stress markers in a murine sepsis model [49]
Underlying mechanisms	‐ High ADM levels likely represent a failing compensatory response, aimed at restoring endothelial barrier functions in sepsis ‐ ADM‐enhancing therapies have potential beneficial (stabilization of the endothelial barrier) and detrimental (vasodilation) effects in sepsis ‐ The non‐neutralizing ADM antibody Adrecizumab enhances ADM’s beneficial effects whilst attenuating its detrimental effects [117, 135]	‐ High cDPP3 levels are caused by uncontrolled release of cytosolic DPP3 in the circulation following cell death ‐ The uncontrolled release of cDPP3 further deteriorates vascular tone by inhibiting compensatory angiotensin II responses

ADM, adrenomedullin; DPP3, dipeptidyl peptidase 3; cDPP3, circulating dipeptidyl peptidase 3; VSMC, vascular smooth muscle cell.

## DPP3 administration and DPP3 antibodies

In a murine model of hypertension induced by continuous infusion of angiotensin II by an implanted micro‐osmotic pump, intravenous administration of DPP3 rapidly normalized blood pressure to a similar extent as the angiotensin receptor blocker candesartan [21]. Prolonged DPP3 infusion also ameliorated the development of cardiac hypertrophy and fibrosis in these hypertensive mice, and reduced urinary albumin excretion and markers of kidney injury [21]. Of note, DPP3 infusion reduced circulating angiotensin II levels to even lower levels than baseline, suggesting that endogenously produced angiotensin II was also effectively cleaved. In DPP3 (−/−) knockout mice, significant upregulation of the classical RAAS was observed, reflected by higher circulating levels of angiotensin II, angiotensin III, angiotensin IV and angiotensin 1‐5, all known substrates of DPP3 [17].

In healthy mice, DPP3 administration provoked rapid deterioration of left ventricular function, as well as increased renal resistance indexes. Following cessation of DPP3 administration, both left ventricular function and cDPP3 levels returned to pre‐infusion levels within approximately 120 min [46]. Additionally, high cDPP3 levels were observed in a murine isoproterenol heart failure model, which were associated with reduced shortening fraction, high resistive renal index and pulmonary congestion [46].

Although much more limited, there are also data on antagonizing DPP3. Interestingly, in the above‐mentioned heart failure model, administration of a neutralizing DPP3 antibody normalized left ventricular function, an effect which was sustained after 24 h and 14 days [46]. Furthermore, administration of the same DPP3 antibody in murine sepsis attenuated sepsis‐induced cardiac dysfunction and improved overall survival [49]. Following the recent findings in these animal models, DPP3 inhibitors are currently being developed for clinical use in septic and cardiogenic shock patients [46]. An overview of the effects of cDPP3‐modulating therapies is presented in Table 1.

## Adrenomedullin

ADM is a freely circulating 52‐amino acid peptide, first isolated from human pheochromocytoma tissue more than two decades ago [50]. The formation of biologically active ADM is preceded by a multistep cleavage process. First, a 21‐residue N‐terminal signalling peptide is cleaved of the 185‐amino acid‐long preprohormone (prepro‐ADM), resulting in a 164‐amino acid peptide called pro‐ADM. Pro‐ADM is subsequently cleaved into different fragments, including pro‐ADM N‐terminal 20 peptide (PAMP) [51, 52, 53], midregional pro‐ADM (MR‐pro‐ADM) [54], adrenotensin [55] and a glycine‐extended 53‐amino acid peptide. This last peptide is converted to biologically active ADM through subsequent enzymatic amidation [56]. Although initial studies primarily identified vasodilatory properties of ADM, a myriad of biological functions have since been discovered. Genetic evidence points to the protection of the endothelial barrier as the key function of ADM *in vivo* [57]. ADM exerts these effects through binding to the ADM1 and ADM2 receptors, heterodimeric complexes consisting of the calcitonin receptor‐like receptor (CRLR) and specific receptor activity‐modifying proteins (RAMP)2 and RAMP3, respectively [58].

ADM is ubiquitously expressed in almost all human tissues [25], with highest ADM concentrations found in the adrenal medullae, cardiac atria and lungs [59, 60]. ADM is produced by multiple cell types including endothelial cells, VSMCs, macrophages, monocytes and renal parenchymal cells [61, 62, 63, 64, 65, 66]. Similar to the ubiquitous expression of the ADM peptide, ADM receptors are also present in multiple tissues including blood vessels, heart, lungs, skeletal muscles and nerve tissues [67, 68, 69, 70].

Studies in rat endothelial cell lines showed that ADM is not stored, but constantly produced. Moreover, it was shown that endothelial cells secrete ADM at a higher rate than VSMCs [71]. ADM has a short half‐life of approximately 22 min [72]. Degradation occurs through cleavage of its N‐terminus by different circulating and membrane‐bound proteases, of which neprilysin is the most important [73, 74, 75]. ADM is also degraded through internalization and degradation of activated ADM receptor complexes, with the lungs being involved as major site of clearance [76, 77, 78].

## Adrenomedullin as a regulator of vascular tone

The first discovered effect of ADM was vasodilation, causing reduced peripheral resistance and hypotension in animal studies [50, 79]. Following these initial findings, *ex vivo* studies demonstrated the direct vasodilatory effects of ADM in isolated blood vessels and isolated organs [80, 81, 82]. Concurrently, *in vivo* studies in both animals and humans showed that intravenous infusion of ADM decreased blood pressure and induced a compensatory increase in heart rate, as well as enhanced endogenous noradrenaline and renin concentrations, and increased cardiac output [79, 83, 84, 85, 86, 87, 88].

ADM mediates its vasodilatory effects through binding with its target receptors on endothelial cells and VSMCs [80]. Both endothelium‐dependent and endothelium‐independent signalling pathways are implied in ADM’s vasodilatory effects on VSMCs [89]. The latter are caused by direct binding of ADM to its receptors on VSMCs, which leads to increased cyclic adenosine monophosphate (cAMP) and smooth muscle cell relaxation [90, 91]. Several endothelium‐dependent pathways through which ADM causes vasodilatation are all mediated by increased endothelial nitric oxide synthase (eNOS) activity, leading to local nitric oxide (NO) release and consequent vasorelaxation [92, 93].

ADM has also been implicated in central regulation of blood pressure, although studies have yielded contradictive findings. The presence of endogenous ADM in the hypothalamus has been demonstrated [94], and microinjections of ADM into the hypothalamic paraventricular nucleus elicited an immediate and short‐lived decrease in blood pressure in animal studies [95, 96]. In contrast, infusion of ADM directly into intracerebral fluid and microinjections of ADM in the rostral ventrolateral medullae were both found to increase blood pressure in different animal studies [97, 98].

## Adrenomedullin as a regulator of endothelial barrier function

The single‐cell layer of vascular endothelium in blood vessels constitutes the barrier between the intravascular and interstitial spaces. The endothelium is essential in regulating the diffusion of molecules and other substrates through paracellular and transcellular transport mechanisms [99, 100]. Because of its location, the endothelium also fulfils unique regulatory functions on local vessel tone, local and systemic inflammatory signalling, and haemostasis and angiogenesis [8, 100, 101].

Following injury, inflammation causes barrier compromise at the endothelial cell‐to‐cell junction level, subsequently allowing for the efflux of inflammatory signal molecules (e.g. cytokines and prostaglandins) and leucocyte infiltration into tissues [8]. These processes are physiological responses paramount to fight off infection locally, as they are required to combat pathogens residing in the tissues. Nevertheless, during sepsis, excessive systemic damage to the endothelial barrier induced by the inflammatory response causes large amounts of intravascular fluids to leak into tissues, leading to oedema formation, which substantially contributes to the development of shock [4, 102].

ADM is essential for endothelial barrier development and maintenance [19]. Knockout mice lacking crucial parts of ADM‐ADM receptor signalling pathways develop lethal hydrops fetalis, indicating inadequate development of the endothelial barrier [103, 104, 105]. In conditional murine knockout models, in which either ADM synthesis by endothelial cells or endothelial ADM receptors were defective, increased vascular permeability and oedema formation were observed [106, 107], further illustrating the relevance of this pathway.

*In vitro* studies have demonstrated that ADM stabilizes the endothelial barrier through regulation of the actin–myosin cytoskeleton [108]. In cultured human umbilical vein endothelial cells and porcine pulmonary artery endothelial cell monolayers, pretreatment with ADM reduced endothelial hyperpermeability elicited by hydrogen peroxide (H_2_O_2_), thrombin and haemolysin A, by attenuating myosin light‐chain phosphorylation, stress fibre formation and subsequent gap formation through a cAMP‐dependent mechanism [109]. ADM also diminished H_2_O_2_‐induced oedema formation in isolated perfused rabbit lungs, accompanied by increased cAMP levels in the lung perfusate [109].

## Adrenomedullin as a treatment target relevant for sepsis

Circulating ADM levels were found to have prognostic value for clinical outcome in a range of pathophysiological conditions. Whilst various studies have described high ADM levels in patients with congestive heart failure, acute heart failure, cardiogenic shock and sepsis, the highest concentrations are found in patients with septic shock [110, 111, 112, 113, 114]. In septic shock patients, ADM levels correlate with disease severity, mortality and different types of organ dysfunction, including vasopressor/inotrope dependency and need for renal replacement therapy [110, 111, 112, 115, 116]. Moreover, a reduction in ADM following the first day of treatment in the ICU was associated with improvements in organ dysfunction scores and lower 28‐day mortality [116].

Whilst these associations might suggest that ADM plays a detrimental role in sepsis and that neutralizing ADM may be beneficial, no causal relationships should be deducted because of the observational nature of these studies. In the light of the beneficial effects of ADM on endothelial barrier function, the increase in ADM likely represents a failing compensatory response, aimed to protect against inflammation‐induced organ damage in sepsis [117]. Over the last decades, several studies have investigated the effects of ADM administration or other ADM‐targeted therapies in preclinical models of sepsis. Different approaches were used, including ADM administration, modulation of ADM function, and partial or complete neutralization using anti‐ADM antibodies. In this respect, it is important to note that ADM‐related therapies have previously been described as ‘a double‐edged sword’ in sepsis [118]. As mentioned before, apart from stabilization of the endothelial barrier, ADM also has the potential to cause vasodilatation and hypotension, which may contribute to worse outcome of septic shock patients [108, 109, 119, 120, 121]. As such, the beneficial effects of ADM on vascular permeability on the one hand and possible detrimental effects of vasodilatation on the other hand suggest that tight regulation of ADM is required [118]. An overview of the effects of ADM is presented in Fig. 1 and Table 1.

## Adrenomedullin administration

Data on ADM administration during inflammatory conditions are limited to animal models. In various endotoxaemia models, ADM administration resulted in improved haemodynamics and reduced vascular leakage, end‐organ damage and mortality [119, 120, 122, 123, 124]. ADM administration also attenuated kidney injury in two different renal injury models [125, 126]. In lung injury models, ADM administration reduced endothelial hyperpermeability, histopathological features and levels of pro‐inflammatory cytokines [121, 127, 128]. Lastly, in an *in vivo* murine model of shock induced by injection of *S. aureus* alpha toxins, ADM administration reduced albumin and plasma fluid extravasation, and improved survival [108]. Of note, all the aforementioned studies on ADM were performed using either endotoxaemia or organ injury models, which do not necessarily recapitulate infection. There are also several drawbacks to direct administration of ADM in sepsis and septic shock, limiting its clinical applicability. Because of the aforementioned short half‐life of ADM of only 22 min [72], ADM therapy would have to be applied as prolonged continuous intravenous infusion. Increasing ADM’s half‐life through PEGylation may circumvent this issue [129], but no data on the effects of PEGylated ADM in animal models of sepsis are currently available. Even more important, as ADM possesses potent vasodilatory effects, ADM administration might well induce hypotension in a patient category already at great risk of hypotension‐induced end‐organ failure [118]. An overview of the effects of ADM‐modulating therapies is presented in Table 1.

## Adrenomedullin‐binding antibodies

ADM interacts with its receptor through its C‐terminal moiety [130], whilst the N‐terminal part of ADM is thought to be of only minor importance for its agonist function. In preclinical animal studies, several high‐affinity mouse monoclonal anti‐ADM antibodies have been developed, targeting different epitopes of ADM [131]. Interestingly, complete inhibition of ADM signalling by an antibody targeting the C‐terminus of ADM did not improve survival in murine caecal ligation and puncture (CLP) models of sepsis [131], whilst an antibody targeted at the N‐terminus of ADM (HAM1101), which only results in marginal loss of ADM signalling, resulted in a substantial reduction in mortality in the same sepsis model [131]. Subsequent experiments in CLP‐induced sepsis models also demonstrated that this antibody decreased iNOS, but not eNOS expression, reduced catecholamine infusion rates, attenuated kidney dysfunction and improved survival [132, 133]. This partially inhibitory ADM antibody was subsequently humanized (HAM8101) for use in follow‐up human studies and was named Adrecizumab [117].

The proposed mechanism of action through which Adrecizumab exerts its beneficial effects is of special interest; an overview is presented in Fig. 1. In both preclinical models of sepsis and sepsis patients, Adrecizumab causes a potent, dose‐dependent increase in circulating bioactive ADM [133, 134]. This increase in circulating bioactive ADM levels is not caused by increased synthesis, as concentrations of MR‐pro‐ADM (an inactive peptide fragment derived from the same prohormone as ADM) remained unchanged [134]. It is assumed that modulation of the ADM equilibrium between the blood and interstitial compartments accounts for the increase in ADM following administration of Adrecizumab. ADM is a small peptide molecule that can freely cross the endothelial barrier, whilst the large molecular weight of Adrecizumab precludes its free diffusion [117, 134] and remains in the circulation. Subsequently, binding of circulating ADM to the antibody may drain ADM from the interstitial space, by effectively trapping it in the blood compartment. Moreover, antibody binding also increases the half‐life of ADM, likely by limiting its hydrolysis [117, 134, 135]. Despite the partial inhibition of ADM signalling function caused by antibody binding, this is overruled by the much larger increase in circulating bioactive ADM concentrations resulting in an overall increase in ADM activity in the blood compartment. Being confined to the circulation, ADM exerts its beneficial effect on endothelial cell barrier function, whilst the detrimental vasodilatory effects on VSMCs in the interstitial space are reduced [117]. A more detailed description of the proposed mechanism of action of Adrecizumab is provided elsewhere [135].

## The Adrenomedullin and Outcome in Sepsis and Septic Shock (AdrenOSS) trials as an example of biomarker‐driven sepsis trial design

Thus far, the typical phase 2 and phase 3 clinical sepsis trial design consisted of enrolling sepsis patients fitting broad inclusion criteria (e.g. current sepsis definitions) not taking into account whether the biological pathways influenced by the specific treatment are activated or inhibited in a specific patient [14, 136]. The use of these broad criteria results in marked population heterogeneity with a large noise‐to‐signal ratio, leading to smaller chances to detect treatment effects even when sample sizes are increased [137]. Consequently, large amounts of resources have gone into studies with limited chances to detect any clinically relevant treatment effects [137, 138, 139].

**Fig. 2:**
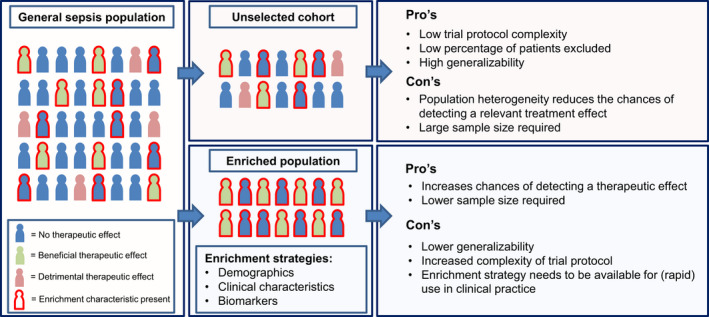
Concept of population enrichment in sepsis trial design. The enrichment characteristic is related to the mode of action of the treatment under study. This can constitute demographic features, clinical characteristics, elevated/depressed biomarkers or a combination of these.

Population enrichment strategies consist of the preselection of a study population based on patient characteristics specifically associated with the biological pathway modulated by the investigational treatment [136]. Candidate characteristics for population enrichment include the use of biomarkers, imaging or clinical characteristics that correlate with certain disease phenotypes [137, 140]. Because preselecting a population based on biological responses related to the intervention will increase the chance of a trial to detect a treatment effect, it may allow for smaller sample sizes than would be required in unselected populations [137]. This tailoring of trial design to include only a subgroup of patients most likely to benefit from the treatment instead of a ‘one‐drug‐fits‐all’ model is known as precision medicine [137, 141]. Whilst the call for precision medicine in sepsis trial design has been around for more than a decade following the failure of many phase 2 and phase 3 clinical trials, examples of studies actually incorporating these design features have been extremely sparse [138, 139, 141]. A graphic overview of the concept of population enrichment strategies is provided in Fig. 2.

Following phase 1 studies demonstrating a favourable safety and tolerability profile of Adrecizumab [134], design of a follow‐up phase‐2 ‘proof‐of‐concept’ trial in septic shock was initiated. This ‘AdrenOSS‐2’ study represents one of the first examples of a sepsis trial incorporating the use of a biomarker as an enrichment strategy. For AdrenOSS‐2, patients more likely to experience adverse outcome caused by endothelial dysfunction were selected for therapy with Adrecizumab using a biomarker‐driven approach incorporating bedside measurements of bioactive ADM (bio‐ADM, SphingoTec GmbH). This biomarker approach was chosen based on the concept of high ADM levels as a physiological response to maintain endothelial barrier integrity that falls short during sepsis [117, 135].

In order to decide on a specific cut‐off level of bio‐ADM, which would serve as an inclusion criterium, the relationship between initial levels of bio‐ADM and short‐term outcome in sepsis and septic shock patients was first examined in a prospective multicenter cohort study called ‘AdrenOSS‐1’. In this study, serial determinations of bio‐ADM defined a cut‐off value of >70 pg mL^−1^ as the best predictor of subsequent organ dysfunction and 28‐day mortality [110, 115, 116]. This cut‐off value was selected as an additional inclusion criterium for the AdrenOSS‐2 trial. Moreover, the measurements of other biomarkers (including cDPP3) were also performed in the trial, to examine whether additional population enrichment using these biomarkers would be able to improve future sepsis trial design.

**Fig. 3:**
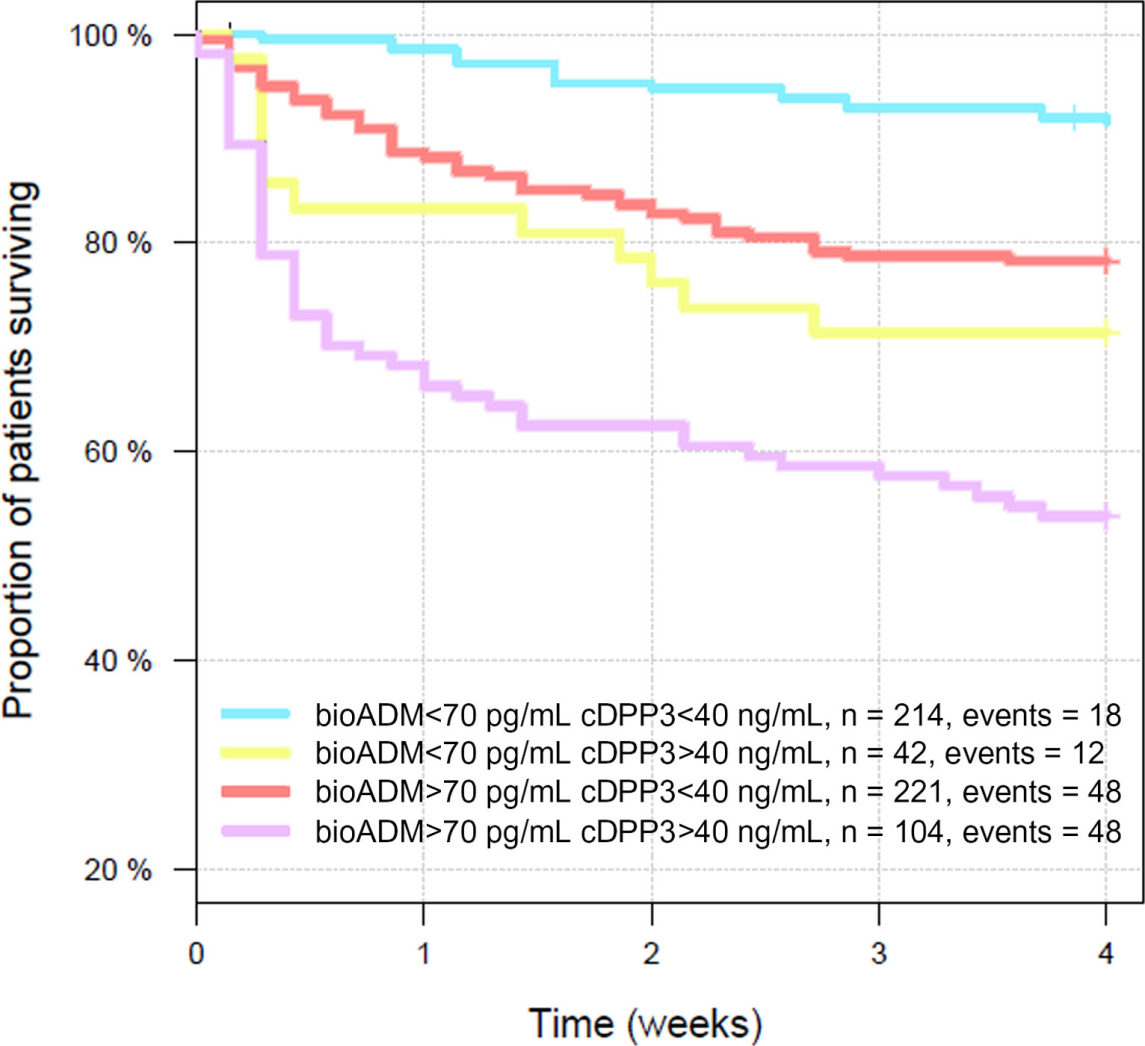
Kaplan–Meier analysis of 28‐day all‐cause mortality in septic shock patients included in the observational AdrenOSS‐1 study. Across the study, 38% of patients displayed elevated bio‐ADM levels, 7% elevated cDPP3 levels, 18% elevated levels of both biomarkers, and 37% elevated low levels of both biomarkers. Bio‐ADM = bioactive adrenomedullin. cDPP3 = circulating dipeptidyl peptidase 3.

Interestingly, whilst preliminary results of AdrenOSS‐2 presented at the 40th International Symposium on Intensive Care & Emergency Medicine demonstrated beneficial treatment effects of Adrecizumab, including an early reduction in organ dysfunction scores, these treatment effects became more pronounced when patients who also exhibited high pretreatment levels of cDPP3 were excluded [142]. As alluded to before, the uncontrolled release of cytosolic DPP3 into the circulation caused by cellular necrosis during shock leads to decreased vascular tone through inhibition of compensatory angiotensin II responses. This represents a biological pathway, which is not targeted by Adrecizumab. Therefore, when patients with high cDPP3 levels were excluded from the analysis, more pronounced beneficial treatment effects for Adrecizumab were found [142]. These findings are also supported by results from the AdrenOSS‐1 study indicating that bio‐ADM and cDPP3 are independent predictors of mortality in sepsis (unpublished data). In this observational study, both biomarkers combined improved the c‐index for 28‐day mortality to 0.742, whereas it was 0.688 for bio‐ADM and 0.692 for cDPP3 alone (p‐value for added value < 0.0001) (unpublished data). In patients with bio‐ADM < 70 pg mL^−1^, 16% had elevated cDPP3 (>40 ng mL^−1^, upper normal range). These patients had a substantially worse outcome (HR: 3.9, 95% confidence interval [CI]: 1.9–8.1, 28‐day survival rate: 71%) compared to patients with both low cDPP3 and low bioactive ADM (28‐day survival rate: 92%). Patients with elevated bio‐ADM but normal cDPP3 (constituting 68% of patients with elevated bio‐ADM) also had a worse outcome than patients with low values in both biomarkers (HR: 2.8, 95% CI: 1.6–4.8, 28‐day survival rate: 78%). Importantly, patients with the highest fatality rate were those who displayed elevated levels of both biomarkers (HR: 7.4, 95% CI: 4.3–12.8; 28‐day survival rate: 54%; reference group: patients who had low levels of both biomarkers) (unpublished data). The Kaplan–Meier curves for these respective groups are displayed in Fig. 3.

These results provide further evidence that ADM and DPP3 represent two distinct pathways involved in the development of organ dysfunction in sepsis and that enrichment strategies combining these biomarkers may improve the therapeutic benefit of therapies targeting ADM and DPP3‐specific pathways.

## Conclusion

DPP3 is a ubiquitous, primarily cytosolic enzyme involved in the degradation of several important signal molecules relevant for the regulation of vascular tone, including angiotensin II. ADM is a key hormone involved in the regulation of vascular tone and endothelial barrier function. Increased release of these molecules during sepsis relates to vascular tone and capillary leakage, both independently associated with the development of organ failure and adverse outcome in sepsis. Therefore, these molecules likely represent two unique and distinct pathways of clinical significance involved in the development of septic shock. Both ADM‐enhancing therapies aimed at improving endothelial barrier function and DPP3‐blocking therapies aimed at restoring systemic angiotensin responses have been shown to improve outcome in various preclinical sepsis models. Given the availability of rapid bedside biomarker assays for both DPP3 and ADM, they represent promising opportunities for the conduct of biomarker‐guided sepsis trials. Given the current lack of any adjuvant therapy in sepsis, additional research on the therapeutic application of these peptides in humans is highly warranted.

## Conflict of Interest statement

PP received travel and consultancy reimbursement from Adrenomed and 4TEEN4, the companies that produced the ADM and DPP3 bio‐assays and antibodies described. The other authors declare no financial conflicts of interest.

## Author contribution

**Dirk van Lier:** Conceptualization (supporting); Writing‐original draft (lead). **Matthijs Kox:** Conceptualization (supporting); Writing‐review & editing (supporting). **Peter Pickkers:** Conceptualization (lead); Writing‐review & editing (lead).
